# Elicitation of Expert Prior Opinion: Application to the MYPAN Trial in Childhood Polyarteritis Nodosa

**DOI:** 10.1371/journal.pone.0120981

**Published:** 2015-03-30

**Authors:** Lisa V. Hampson, John Whitehead, Despina Eleftheriou, Catrin Tudur-Smith, Rachel Jones, David Jayne, Helen Hickey, Michael W. Beresford, Claudia Bracaglia, Afonso Caldas, Rolando Cimaz, Joke Dehoorne, Pavla Dolezalova, Mark Friswell, Marija Jelusic, Stephen D. Marks, Neil Martin, Anne-Marie McMahon, Joachim Peitz, Annet van Royen-Kerkhof, Oguz Soylemezoglu, Paul A. Brogan

**Affiliations:** 1 Department of Mathematics and Statistics, Lancaster University, Lancaster, United Kingdom; 2 Department of Rheumatology, UCL Institute of Child Health, London, United Kingdom; 3 MRC North West Hub for Trials Methodology Research, Department of Biostatistics, University of Liverpool, Liverpool, United Kingdom; 4 Department of Renal Medicine, Addenbrooke's Hospital, Hills Road, Cambridge, United Kingdom; 5 Department of Medicine, University of Cambridge, Cambridge, United Kingdom; 6 Medicines for Children Research Network Clinical Trials Unit, University of Liverpool, Alder Hey Children’s NHS Foundation Trust, Liverpool, United Kingdom; 7 Department of Women's & Children's Health, Institute of Translational Medicine, University of Liverpool, Liverpool, United Kingdom; 8 Division of Rheumatology, Department of Paediatric Medicine, IRCCS Bambino Gesù Children Hospital, Piazza Sant’Onofrio 4, 00165, Rome, Italy; 9 Oporto Medical School, Integrated Hospital S. João, Porto, Portugal; 10 AOU Meyer, Viale Pieraccini 24, 50139, Florence, Italy; 11 Department of Pediatric Rheumatology and Nephrology, Universitary Hospital Ghent, Ghent, Belgium; 12 Department of Paediatrics and Adolescent Medicine, Charles University in Prague, 1st Faculty of Medicine and General University Hospital, Prague, Czech Republic; 13 Great North Children’s Hospital, Newcastle upon Tyne, United Kingdom; 14 Department of Paediatric Rheumatology, University of Zagreb School of Medicine, University Hospital Centre Zagreb, Zagreb, Croatia; 15 Department of Paediatric Nephrology, Great Ormond Street Hospital for Children NHS Foundation Trust, Great Ormond Street, London, WC1N 3JH, United Kingdom; 16 Department of Paediatric Rheumatology, Yorkhill Hospital, Glasgow, United Kingdom; 17 Sheffield Children’s Hospital NHS Foundation Trust, Sheffield, United Kingdom; 18 Hospital for Children and Adolescents, University Hospital of Cologne, Cologne, Germany; 19 Wilhelmina Children’s Hospital, University Medical Centre, Utrecht, Netherlands; 20 Department of Pediatric Nephrology and Rheumatology, Gazi University Hospital, Ankara, Turkey; Politecnico di Torino, ITALY

## Abstract

**Objectives:**

Definitive sample sizes for clinical trials in rare diseases are usually infeasible. Bayesian methodology can be used to maximise what is learnt from clinical trials in these circumstances. We elicited expert prior opinion for a future Bayesian randomised controlled trial for a rare inflammatory paediatric disease, polyarteritis nodosa (MYPAN, Mycophenolate mofetil for polyarteritis nodosa).

**Methods:**

A Bayesian prior elicitation meeting was convened. Opinion was sought on the probability that a patient in the MYPAN trial treated with cyclophosphamide would achieve disease remission within 6-months, and on the relative efficacies of mycophenolate mofetil and cyclophosphamide. Expert opinion was combined with previously unseen data from a recently completed randomised controlled trial in ANCA associated vasculitis.

**Results:**

A pan-European group of fifteen experts participated in the elicitation meeting. Consensus expert prior opinion was that the most likely rates of disease remission within 6 months on cyclophosphamide or mycophenolate mofetil were 74% and 71%, respectively. This prior opinion will now be taken forward and will be modified to formulate a Bayesian posterior opinion once the MYPAN trial data from 40 patients randomised 1:1 to either CYC or MMF become available.

**Conclusions:**

We suggest that the methodological template we propose could be applied to trial design for other rare diseases.

## Introduction

Recently, the European Commission and subsequently the UK Department of Health prioritised strategies to improve the care of patients with rare diseases (prevalence ≤ 5 per 10,000) [1]. Such strategies should consider alternative therapeutic trial designs for small anticipated sample sizes [2]. The Bayesian approach begins by formally characterising prior opinion which is then updated with collected data using Bayes theorem to obtain a posterior opinion to inform clinical practice. It can be particularly useful when the sample sizes required by traditional frequentist designs are infeasible [2, 3]. But what is a Bayesian prior? And how do you elicit one? Accounts have been given of Bayesian prior elicitation in the context of designing randomised controlled trials [4–5] (RCTs). The findings of a Bayesian prior elicitation exercise have been cited to motivate conduct of an RCT evaluating warfarin for the treatment of rheumatic diseases [6]. However, such methods have yet to become part of routine practice for paediatric trials. This paper describes the novel approach that was taken to elicit expert prior opinion to inform the design of an RCT for a rare disease affecting children, polyarteritis nodosa (PAN). A full account of the statistical aspects of the proposed approach is given elsewhere [7]. The aim of the current paper is to provide further practical details on the steps taken to plan and carry out the Bayesian prior elicitation meeting so this process can be replicated by clinical trialists in other rare disease studies.

### Polyarteritis nodosa and the MYPAN trial

Childhood PAN is a rare and severe multi-systemic vasculitic disease that affects approximately 1 per million children [8]; a full clinical description is provided elsewhere [8, 9]. Untreated, mortality is close to 100% [10]; with aggressive immunosuppression mortality is 4% [9]. A comprehensive literature search of published and unpublished studies relating to the treatment of PAN was undertaken (full details of the search strategy and databases searched are available upon request); an important finding was that there are no published or currently recruiting RCTs relating to PAN in children. All of the paediatric reports identified were uncontrolled study designs i.e. cohort and single-case studies, that rate as low-level evidence. Conclusions based on RCTs in adults with PAN [11–14] with important implications for children are: a) treatment of severe PAN requires corticosteroids combined with intravenous cyclophosphamide (CYC); b) despite therapy, mortality associated with PAN in adults remains high at 4–22%; treatment-related toxicity contributes to this; c) adverse events (disease and treatment-related) affect 54–100% patients; and d) avoidance of CYC in children is desirable if alternatives exist since complications associated with CYC include infertility and malignancy.

The MYPAN study (Mycophenolate mofetil for childhood PAN) is an open-label non-inferiority RCT of mycophenolate mofetil (MMF) versus CYC for the treatment of PAN in children (4–18 years), currently being set up. Table 1 compares the design of the MYPAN trial with that of the MYCYC trial, an RCT comparing MMF with CYC for remission induction in ANCA-associated vasculitis. The primary endpoint of the MYPAN study is disease remission within six months of randomisation using a standard definition [15]. Due to its lower risk of infertility/malignancy, MMF would be favoured [16] *unless* the 6-month remission rate on MMF was more than 10% (absolute difference) smaller than that on CYC. A definitive frequentist trial would require 513 patients per arm to have 90% power to declare MMF non-inferior to CYC at the 2.5% one-sided significance level when remission rates on both treatments equal 70%. Previous experience suggests it would take well over 30 years to reach this sample size [9, 17], thus explaining why a paediatric trial for PAN has never been performed. Early planning of the MYPAN trial estimated that recruitment of 40 children across 30–40 European centres would be achievable over four years.

**Table 1:** Comparison of the design of 2 randomised controlled trials for vasculitis: MYPAN versus MYCYC.

Trial name and study population	MYPAN^a^: PAN^b^ in children (≥4 and ≤ 18 years)	MYCYC^c^: ANCA^d^ associated vasculitis in adults & children (8 children, 132 adults)
**Hypothesis**	MMF^e^ is not inferior (<10% absolute difference) to intravenous CYC^f^ for induction of remission	MMF is not inferior (<12% absolute difference) to intravenous CYC for induction of remission
**Entry criteria**	i) Must fulfil classification criteria for PAN; ii) 1or more major PVAS^g^ items and/or 3 or more minor PVAS items; iii) Must be newly diagnosed patients	i) Chronic inflammatory disease lasting at least 4 weeks; characteristic histology on biopsy and/or a positive ANCA; ii) 1 or more major BVAS^h^ items and/or 3 or more minor PVAS items; iii) Must be newly diagnosed patients
**Primary endpoint**	Remission within 6 months defined as PVAS 0/63 on 2 consecutive readings at least one month apart on protocol steroid taper	Remission within 6 months defined as BVAS 0/63 on 2 consecutive readings at least one month apart on protocol steroid taper

^a^MYPAN: Mycophenolate mofetil for childhood polyarteritis nodosa;

^b^PAN: polyarteritis nodosa;

^c^MYCYC: Mycophenolate mofetil versus cyclophosphamide for ANCA associated vasculitis;

^d^ANCA: anti neutrophil cytoplasmic antibodies;

^e^MMF: Mycophenolate mofetil;

^f^CYC: cyclophosphamide;

^g^PVAS: Paediatric Vasculitis Activity Score;

^h^BVAS: Birmingham Vasculitis Activity Score.

Instead, the MYPAN trial adopts a Bayesian approach, first characterising expert prior opinion about the 6-month remission rate on CYC and the relative benefit of MMF as probability distributions before the trial begins, and then updating these distributions using Bayes theorem once data become available [2]. This paper gives an account of how expert prior opinion was elicited.

## Materials and Methods

### Identifying and inviting clinical experts

A Prior Elicitation Meeting was convened. Paediatric consultants in rheumatology, nephrology, immunology or other allied specialties were sought from across the UK and internationally, with an interest in vasculitis and experience of looking after children with PAN (having seen on average at least one case every two years). Invitations were sent to society email lists for the Pediatric Rheumatology International Trials Organisation [18] (www.printo.it), the British Society for Paediatric and Adolescent Rheumatology (www.bspar.org.uk), the British Association for Paediatric Nephrology (www.renal.org/BAPN), and the European Society for Pediatric Nephrology (http://espn.cardiff.ac.uk/), and also to 81 paediatric clinics treating PAN identified via Orphanet (www.orpha.net). Initial expressions of interest were received from 25 eligible respondents, of whom (for logistical reasons) 15 were eventually able to attend the meeting. The Expert Group comprised the following co-authors of this manuscript: MWB, CB, AC, RC, JD, PD, DE, MF, MJ, SM, NM, A-MM, JP, A van R-K and OS. Participating experts were drawn from across the EU and Turkey (see author affiliations).

No patients were involved in the Bayesian prior elicitation meeting. Instead, all participants were expert investigators who had volunteered to participate in the meeting (and are co-authors of this manuscript). Since participants were true investigators, rather than subjects involved in research, we did not require ethics approval for the elicitation exercise. We therefore followed the same process as other expert consensus exercises and did not seek written consent.

### Selection of the specific quantities to be elicited

Expert opinion was sought on the probability that a patient satisfying the entry criteria of the MYPAN trial would succeed according to its primary endpoint. The probability of success for a patient treated with CYC was denoted by p_C_, and expert views on the value of p_C_ were elicited directly. The corresponding probability of successful treatment with MMF was denoted by p_M_, the value of which was derived indirectly from expert clinicians using questions about the relative merits of the two drugs. In statistical terms, this relative efficacy was expressed as the log-odds ratio defined by θ = log_e_{p_M_/(1—p_M_)}—log_e_{p_C_/(1—p_C_)}. We interpret θ as a treatment effect, that is, a measure of the advantage of MMF over CYC for improving the chance of disease remission within 6-months. Positive values of θ indicate that the chances of remission are higher on MMF than CYC (superiority); values of θ close to 0 indicate equivalence of efficacy. The non-inferiority margin of δ = 0.1, meaning that MMF would be preferred if p_M_—p_C_ ≥ –0.1, was fixed in advance of the meeting by the clinical trial management group since this difference was felt to be clinically important, and was similar to the definition of non-inferiority used in a previous vasculitis trial (see below and Table 1).

### Mathematical modelling of beliefs and uncertainties

**Fig 1:**
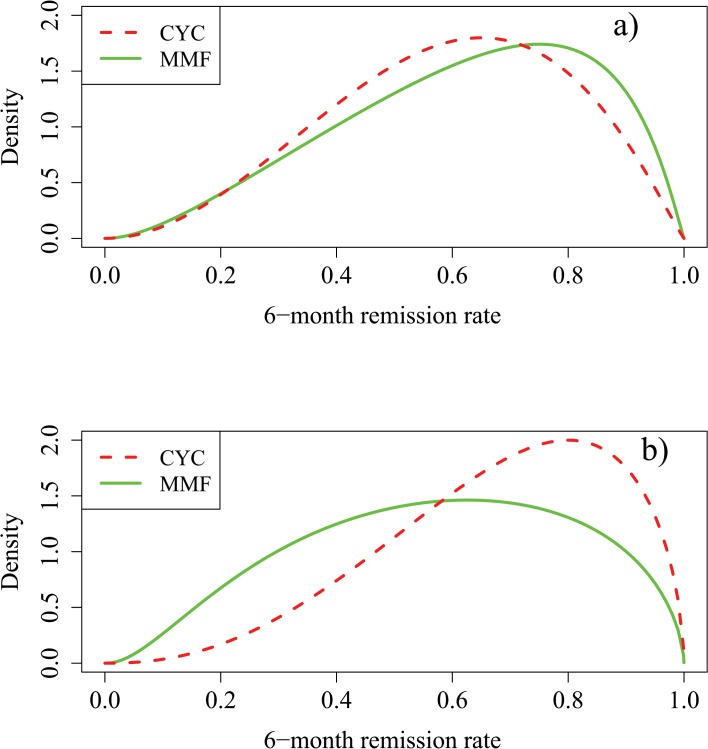
Range of prior opinions elicited before introduction of the MYCYC data. Fig 1A) Comparison of Expert A’s prior densities for p_C_ and p_M_. Expert A thought the most likely value of p_C_ is 0.65 and was 75% confident that p_C_ exceeds 0.45. Expert A was optimistic about the relative efficacy of MMF stating that the chance that MMF is superior to CYC is 63% while the chance it is inferior to CYC is 5%. Fig 1B) Comparison of Expert B’s prior densities for p_C_ and p_M_. Expert B thought the most likely value of p_C_ is 0.8 and was 75% confident that p_C_ exceeds 0.55. Expert B was more sceptical about the benefits of MMF, stating that the chance that MMF is superior to CYC is 10% while the chance it is inferior to CYC is 50%. Given each expert’s prior opinion about p_C_ and the relative efficacies of MMF and CYC, a consistent prior for p_M_ is derived.

Opinion on the value of p_C_ was uniquely defined by two parameters designed to elicit the experts’ choice for the most likely value of p_C_, and their level of uncertainty about this value. These values were sought by asking the experts two questions (Q1 and Q2 in S1 Table). Opinion on the relative efficacy of CYC and MMF (θ) was modelled as a normal distribution, the parameters of which were determined by asking experts two questions (Q3 and Q4 in S1 Table). From the resulting opinions about p_C_ and θ, treated as independent, a consistent opinion about p_M_ was derived. The nature and strength of stated beliefs were characterised through the mean and mode of the distributions of p_C_ and p_M_. To illustrate this, Fig 1 summarises the prior distributions for two experts with extreme opinions about p_C_ and p_M_ relative to the rest of the group. Experts were asked two further questions to verify that the elicited opinion reflected their personal beliefs. Especially useful to the clinicians when considering their prior belief was the calculation of the *effective sample size* [19] (ESS) of a prior distribution; in other words, providing clinicians with the numbers of patients in a hypothetical clinical trial that would be required to statistically generate that level of certainty they had expressed for the parameters. For further details about these calculations, see [7]. The elicitation process was iterative, and the mathematical models and bespoke computer software allowed for quick and clear graphical representations of their densities. The consequences of adding hypothetical data sets to form posterior distributions were also shown. The computer program [7] was written in R [20] using the Shiny package [21] to create a user-friendly, interactive, interface. Software was tested on statistics students (seeking opinions on quantities that they understood) and clinical experts (PB, DE), which led to improvements and modifications.

### Training of the expert participants

**Fig 2:**
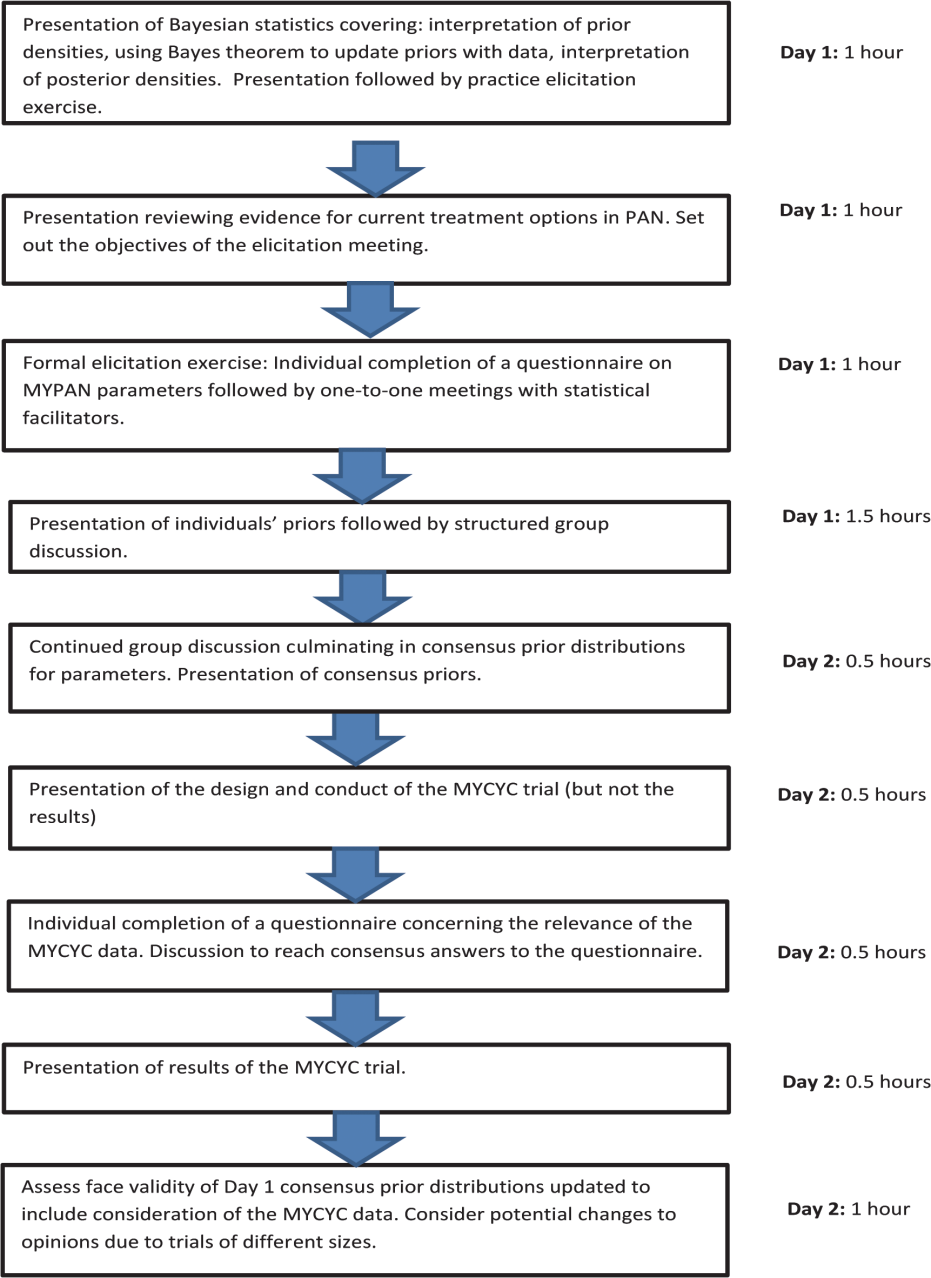
Flow diagram illustrating the sequence of activities undertaken during the MYPAN prior elicitation meeting and the time allocated to each activity.

Fig 2 illustrates the sequence of activities undertaken during the two day meeting and the time allocated to each. Four statistical facilitators were available throughout the two days to support the elicitation process. The meeting began with an overview of PAN [9] and the evidence supporting treatment options including the findings of RCTs in adults [11–14] (S2 Table). A talk by one of the statistical facilitators (JW) introduced Bayesian reasoning, credibility intervals and representation of treatment differences as log-odds ratios. A practice session was then held. A glass jar containing 60 small wooden blocks, coloured either pink or black, was briefly shown to the experts who were then asked for their opinions about the proportion of pink blocks in the jar. Specifically, they were asked for the most likely proportion of pink blocks (interpreted as the most likely value of their prior distribution) and for a value, p_L_, between 0 and 1, which they believed the true proportion of pink blocks exceeded with probability 0.75. Using software similar to that to be used for the real elicitation, opinions were represented through graphics, summaries and ESSs. Having chosen a prior, the wooden blocks were transferred to a black bag, and four volunteers in turn each drew a sample of five, announced the number of pinks, and replaced them. The emerging posterior distribution was displayed. This exercise allowed the principles of the elicitation process and of the Bayesian method to be rehearsed in a neutral setting. The purpose of the statistical training was to standardise the experts’ understanding of Bayesian methods so that they could interpret and check the goodness-of-fit of the prior distributions determined during the formal elicitation process.

### Elicitation of opinions

Each expert was given a structured questionnaire designed to systematically ascertain prior opinion regarding outcomes for treatment with CYC and MMF (S1 File). Experts completed their questionnaires independently, and then met individually with statistical facilitators to discuss their answers. The R program was used to display the consequences of the answers provided. Changes to answers were allowed following discussion with facilitators, until the expert was happy that the model truly reflected their opinion.

Consensus prior distributions for θ and p_C_ were then sought, allowing the group to decide how to weight competing opinions. This was preferred to an automatic mathematical aggregation of individual prior distributions that would require the relevant competencies of participating experts to be quantified, or to working with multiple priors that would lead to multiple posterior distributions and no clear trial conclusion. A nominal group technique process of reaching consensus began with each expert individually commenting to the group on their answers to the structured questionnaire, starting with those with extreme values. Technical misunderstandings were identified and corrected. Lengthy and constructive discussions took place, with the ESS for the opinion about θ being particularly influential in leading to a reduction in the certainty expressed for this parameter. Potential results for the 40 patients in the MYPAN study were considered, and the corresponding posterior distributions displayed to show the relative influence of the prior and the data. Overnight reflection was allowed, and a final consensus (agreed by the majority as reflecting their opinion) was reached on the morning of the second day of the meeting.

### Presentation of related trial results: the MYCYC trial

Once prior distributions had been determined for the parameters of interest, further information was presented to the experts. This concerned the soon-to-be published MYCYC trial (http://www.clinicaltrials.gov/show/NCT00414128) involving 132 adults and 8 children. The design, treatment arms, and primary endpoint for MYCYC were similar to that planned for MYPAN (Table 1), but the final results were not yet published.

### Influence of MYCYC trial results on the prior opinion

Following the presentation of the MYCYC trial design, but before mention of any results, the experts were asked their opinions of the relevance of the MYCYC results to those of MYPAN. Individually they were tasked with completing another structured questionnaire (S2 File) designed to elicit opinion about the relationship between probabilities of response for MYPAN and MYCYC patients on CYC or MMF. Individuals’ answers were displayed on flip charts. Consensus was reached after each expert had explained their views. After consensus had been reached on the relationship between the results of the two trials, key results from MYCYC were presented and key points of comparison between the MYCYC and MYPAN trials were further highlighted. Prior distributions from the first day were then updated by including the influence of the MYCYC results; a full account of the statistical approach used to incorporate these historical data into the prior distributions is provided in [7]. Revisions to opinions about the relationship between the two trials were invited. A final consensus about beliefs in the response probabilities in the MYPAN population was then reached. Opinion about the allocation ratio that should be used in the MYPAN trial was sought but views about the total sample size were not since this number was considered fixed by the maximum feasible number of patients that could be recruited within the timeframe of the trial.

## Results

**Fig 3:**
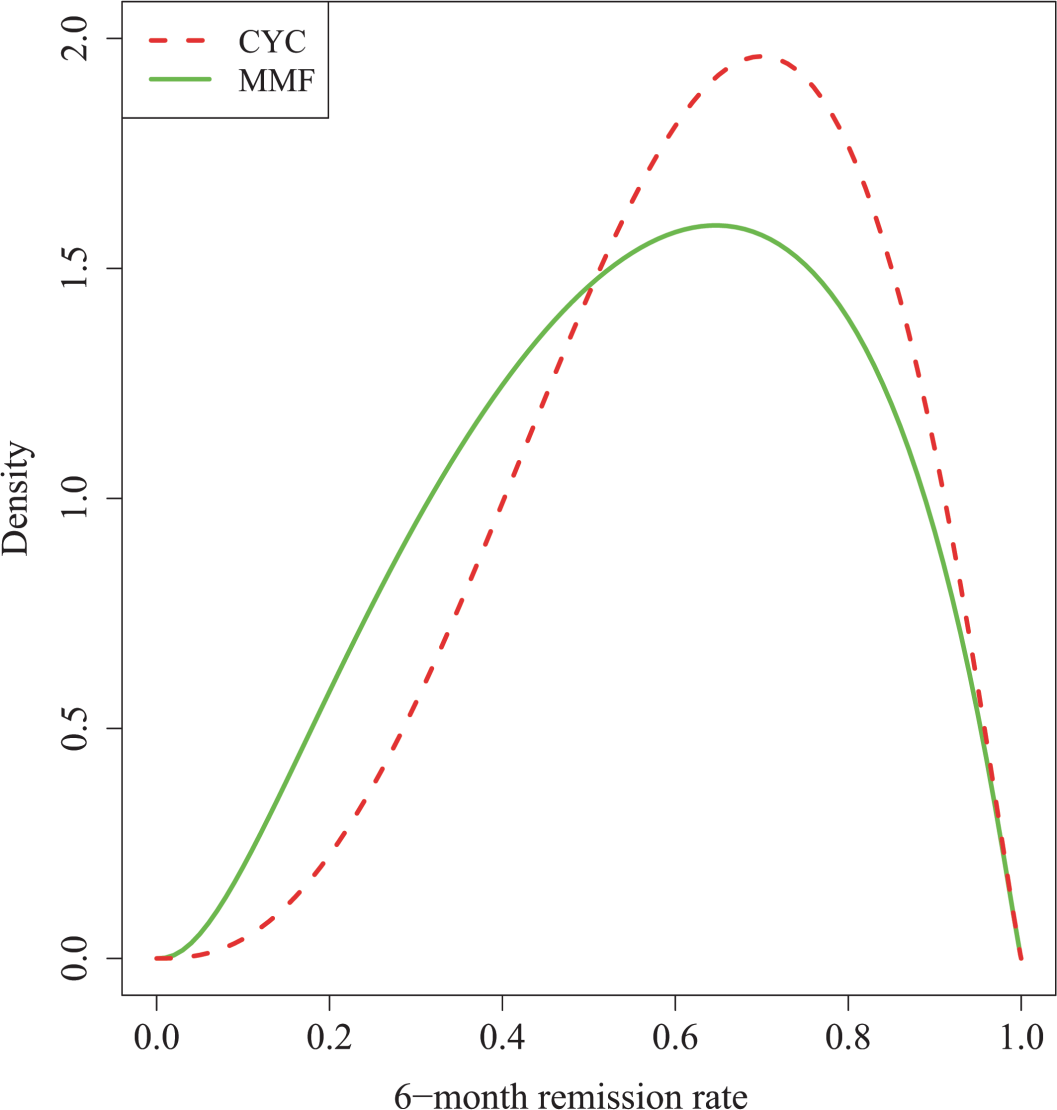
Expert prior opinion before introduction of the MYCYC data regarding 6-month remission rates using treatment with CYC or MMF for children with PAN. **Reprinted from [7] under a CC BY license, with permission from the authors, original copyright 2014.** Prior opinion was that the most likely value for p_C_ was 0.7; 90% and 50% credibility intervals were (0.30, 0.91) and (0.50, 0.78), respectively. The effective sample size was 5 patients on CYC. The prior for p_M_ is derived from those for p_C_ and θ. It had mode = 0.65; 90% and 50% credibility intervals were (0.21, 0.90) and (0.41, 0.74), respectively.

Experts’ final responses to the first structured questionnaire are available online (S1 Table). Group discussion led some experts to change their initial answers, either because they had misinterpreted the questions, or because they accepted suggestions made by their colleagues. Based on the consensus answers of the experts (Fig 3), the most likely value of the remission rate for CYC was 0.70 (90% probability that p_C_ lies between 30% and 91%); this opinion equated to an ESS of 5 patients on CYC. The most likely remission rate on MMF was 0.65 (with 90% probability that p_M_ lies between 21% and 90%). The strength of prior opinion concerning θ corresponded to an ESS of 39 patients on each treatment.

**Fig 4:**
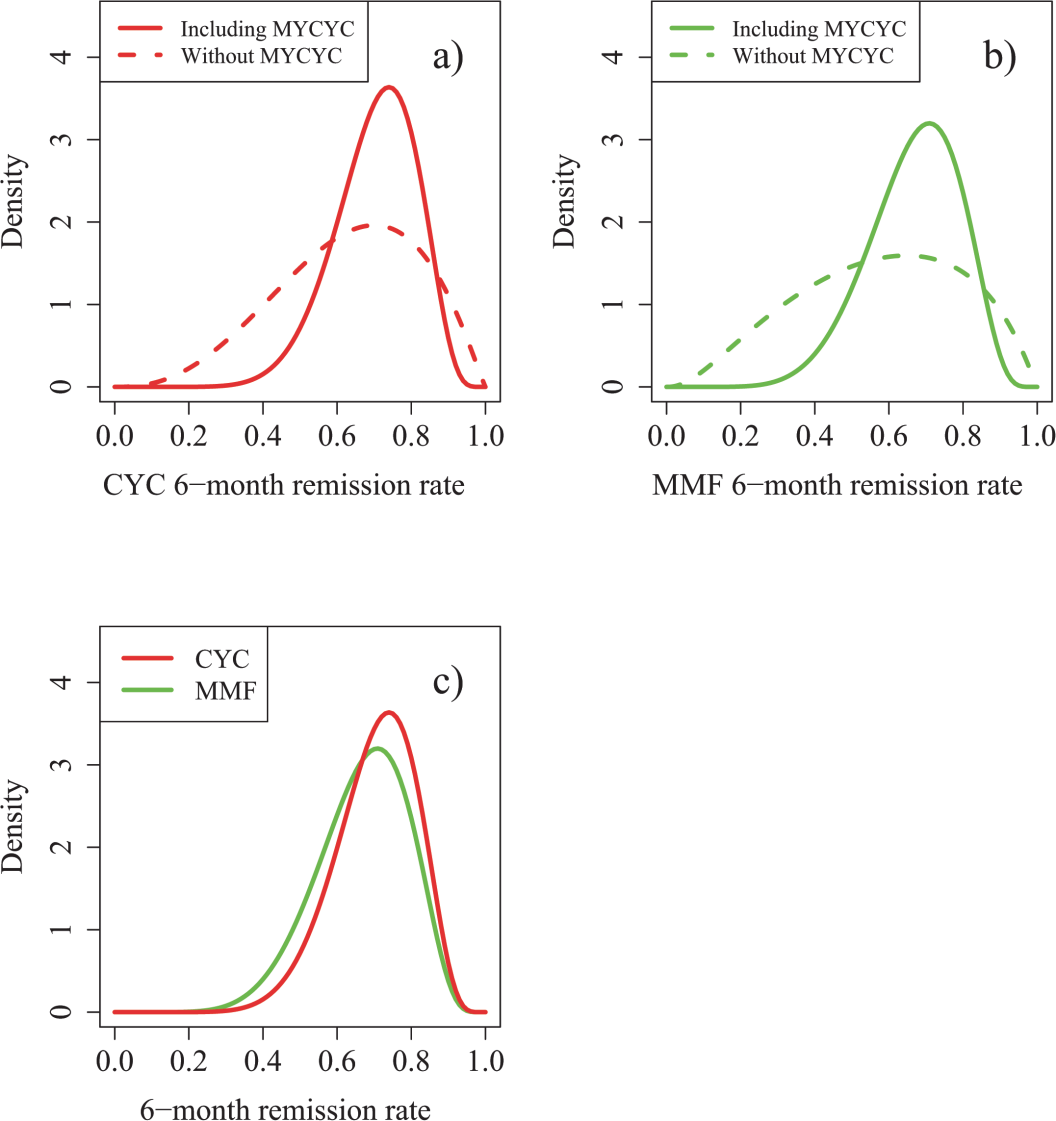
Influence of the MYCYC trial results on expert prior opinion regarding 6-month remission rates using treatment with CYC or MMF for children with PAN. Reprinted from [7] under a CC BY license, with permission from the authors, original copyright 2014. Fig 4A): Influence of MYCYC results on prior opinion for p_C_. The modified prior distribution for p_C_ after considering the MYCYC results had mode = 0.74; 90% and 50% credibility intervals were (0.51, 0.86) and (0.63, 0.78), respectively. This level of certainty is equivalent to what would be obtained from a clinical trial involving 17 patients treated with CYC (effective sample size). Fig 4B): Influence of MYCYC results on prior opinion for p_M_. The modified prior for p_M_ after considering the MYCYC results had mode = 0.71; 90% and 50% credibility intervals were (0.45, 0.85) and (0.59, 0.76), respectively. Fig 4C): Comparison of the final expert prior opinions for p_C_ and p_M_ incorporating the MYCYC data.

**Fig 5:**
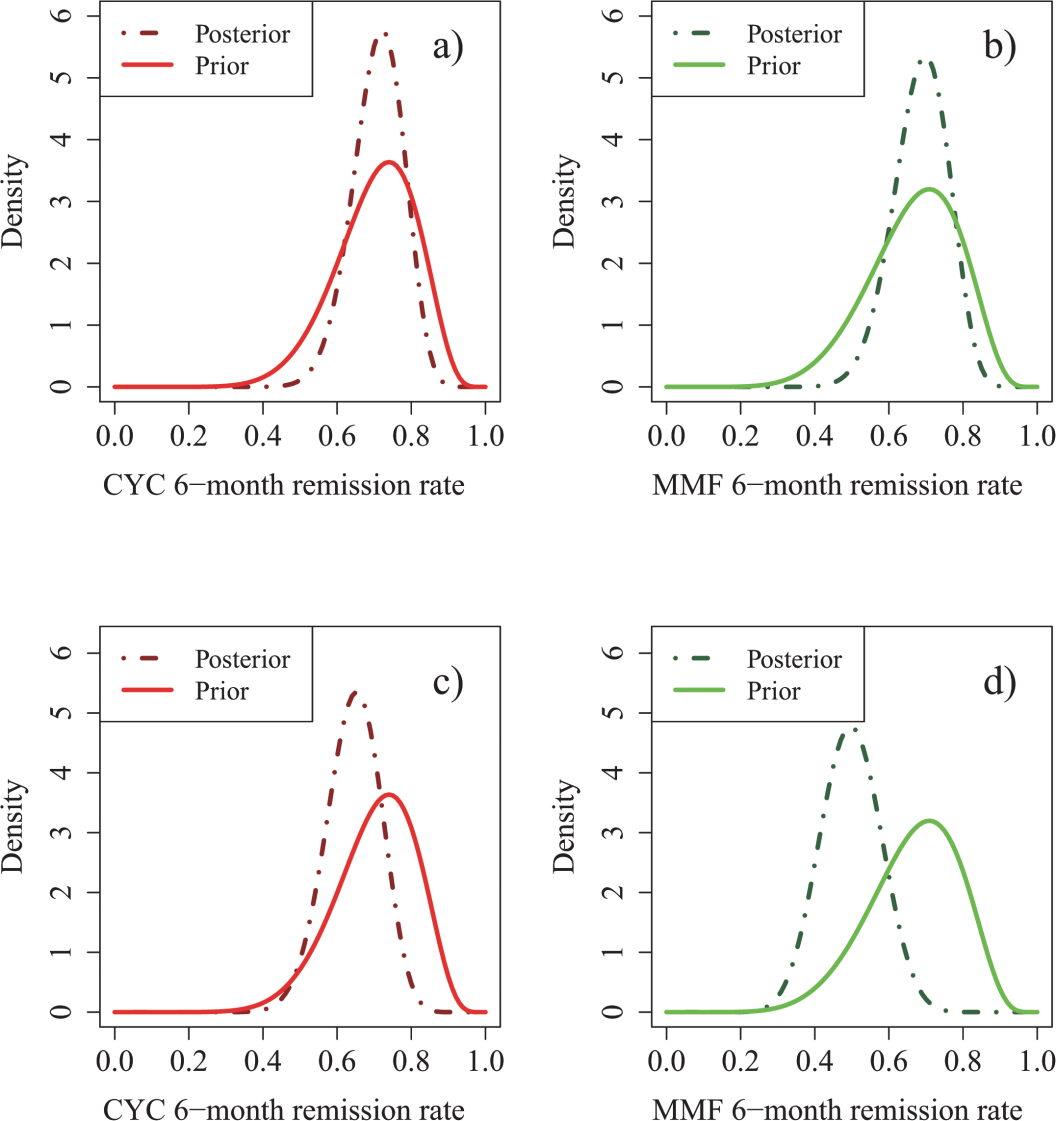
Posterior densities for p_C_ and p_M_ based on the prior of Fig 4 following observation of 20 patients treated on each study arm, with 14/20 successes on CYC and 14/20 successes on MMF (Hypothetical Scenario 1), or with 14/20 successes on CYC and 7/20 successes on MMF (Hypothetical Scenario 2). Fig 5A) and 5C) are reprinted from [7] under a CC BY license, with permission from the authors, original copyright 2014. Fig 5A): Prior and posterior densities for p_C_ in Hypothetical Scenario 1. Fig 5B): Prior and posterior densities for p_M_ in Hypothetical Scenario 1. Fig 5C): Prior and posterior densities for p_C_ in Hypothetical Scenario 2. Fig 5D): Prior and posterior densities for p_M_ in Hypothetical Scenario 2.

The results of the MYCYC trial, in which 70 ANCA associated vasculitis patients were treated with CYC and 70 with MMF, were that 74% of patients on CYC achieved remission within 6 months, compared with 73% of patients on MMF. Incorporating the consensus relevance of these data (S3 Table) resulted in the modified opinions summarised in Fig 4. The most likely remission rates on CYC and MMF changed to 0.74 and 0.71 respectively. In terms of ESS, prior information on p_C_ was now worth 17 patients on CYC and that on θ worth 48 patients on each treatment. The effect of 70 real patients per treatment arm in MYCYC was to increase the former ESS by 12 and the latter by 9, showing how the new information from MYCYC was down-weighted in the context of MYPAN. The MYCYC data had a substantial influence on opinions of the absolute values of remission rates on the two treatments, but much less influence on their relative merits (data not shown). Fig 5 shows how these final prior opinions would change after two hypothetical outcomes of MYPAN itself.

## Discussion

A major challenge in rare diseases is conducting clinical trials with sufficient power to inform best clinical practice when anticipated sample sizes are small. Historically, this has been a major barrier in rare paediatric autoimmune diseases and almost certainly explains why a clinical trial for PAN in children has never been undertaken. We have adopted a Bayesian clinical trial design to overcome this barrier, and describe the process for elicitation of expert prior opinion to inform the design of the MYPAN trial, the first RCT for childhood PAN.

Recently, guiding principles in relation to good practice for prior opinion elicitation have been suggested [22], and where possible we have adopted these. Using a formal Bayesian prior elicitation exercise we have established that the most likely rates of disease remission within 6 months on CYC and MMF are 74% and 71%, respectively. These findings are likely to remain the state of knowledge until they can be updated with data received from MYPAN. Posterior distributions will quantify the uncertainty about disease remission rates that remains once the MYPAN data are available. This uncertainty means that conclusions about whether MMF is non-inferior to CYC are unlikely to be definitive. However, it would take a prospective randomised trial recruiting over 500 patients per arm to achieve that. That said, for a rare disease like PAN, clinically informative results can still be obtained.

Our results revealed that experts can be quite uncertain of absolute effects of treatments, but more convinced of their relative merits. In other words, for MYPAN, experts were uncertain about the precise value of p_C_ but more confident that p_M_ would not be too dissimilar. If the actual data from MYPAN confirm the prior, then confidence in those opinions will grow and this in turn would have an appreciable impact on the treatment approaches that would subsequently be adopted. If the data are at odds with the prior, this will be reflected in a clear and documented change in opinion: the prior is not so strong that contradictory evidence is dismissed.

In the prior elicitation meeting, experts accepted the Bayesian paradigm as a framework for representing their prior knowledge and uncertainty. We canvassed opinion from a pan-European group of experts. However, as the experts were volunteers, their views may not be representative of those who did not accept the invitation to participate.

In conclusion, the methodology developed for this exercise allows formal and structured learning about the treatment of childhood PAN to begin, and to be updated by MYPAN and studies beyond that. We suggest that this methodological template could be applied to trial design for other rare diseases, and is of particular relevance to rare autoimmune conditions that currently lack a good evidence base for treatment.

## Supporting Information

S1 File Structured questionnaire designed to systematically ascertain prior opinion regarding outcomes for treatment with CYC and MMF. (PDF)

S2 File Structured questionnaire designed to ascertain expert opinion on the relevance of the MYCYC results to those of MYPAN. (PDF)

S1 Table Individual experts’ final answers to Q1-Q4 and consensus answers agreed by the group before results from the MYCYC trial were revealed. (PDF)

S2 Table Clinical trials in adults with polyarteritis nodosa. (PDF)

S3 Table Individual experts’ answers to four questions eliciting their beliefs about the relevance of the MYCYC trial results for informing opinion about 6-month remission rates in the MYPAN trial. (PDF)
