# Supplementary material for: Elicitation of Expert Prior Opinion: Application to the MYPAN Trial in Childhood Polyarteritis Nodosa
Source: PLoS One. 2015 Mar 30;10(3):e0120981. doi: 10.1371/journal.pone.0120981 (PMC4378846; doi:10.1371/journal.pone.0120981)
Supplement: S1 File — (PDF) [file pone.0120981.s001.pdf]

S1 File: Structured questionnaire designed to systematically ascertain prior opinion regarding outcomes for treatment with CYC and MMF

NAME:

Before any data are observed, please answer the following questions to specify your prior distributions.

Mark on the scales below your answers to the following questions (to the nearest 0.05).

Q1: What do you think the 6-month remission rate for children with PAN treated with cyclophosphamide (CYC) in combination with corticosteroids (steroids) is?

0 0.1 0.2 0.3 0.4 0.5 0.6 0.7 0.8 0.9 1

Q2: Provide a proportion such that you are 75% sure that the true 6-month remission rate on CYC/steroids exceeds this value.

0 0.1 0.2 0.3 0.4 0.5 0.6 0.7 0.8 0.9 1

Because of the unpleasant side-effects of CYC, mycophenolate mofetil (MMF) might be considered the preferable treatment even if it is associated with a somewhat lower 6-month remission rate:

Q3: What is the chance that the 6-month remission rate on MMF/steroids is higher than that on CYC/steroids?

0 0.1 0.2 0.3 0.4 0.5 0.6 0.7 0.8 0.9 1

Q4: What is the chance that the 6-month remission rate on CYC/steroids exceeds that on MMF/steroids by more than 10%?

0 0.1 0.2 0.3 0.4 0.5 0.6 0.7 0.8 0.9 1

Please answer the following questions which will allow us to check the adequacy of your fitted prior distributions.

Q5: What do you think the 6-month remission rate on MMF/steroids is?

0 0.1 0.2 0.3 0.4 0.5 0.6 0.7 0.8 0.9 1

Q6: Provide a proportion such that you are 75% sure that the true 6-month remission rate on MMF/steroids exceeds this value.

0 0.1 0.2 0.3 0.4 0.5 0.6 0.7 0.8 0.9 1
