# Supplementary material for: Elicitation of Expert Prior Opinion: Application to the MYPAN Trial in Childhood Polyarteritis Nodosa
Source: PLoS One. 2015 Mar 30;10(3):e0120981. doi: 10.1371/journal.pone.0120981 (PMC4378846; doi:10.1371/journal.pone.0120981)
Supplement: S2 File — (PDF) [file pone.0120981.s002.pdf]

S2 File: Structured questionnaire designed to ascertain expert opinion on the relevance of the MYCYC results to those of MYPAN

NAME:

Before the results of the MYCYC trial are revealed, please answer the following questions. We wish to elicit your prior beliefs about the relevance of the MYCYC data for informing opinion about the efficacy of MMF/steroids and CYC/steroids in the MYPAN trial population.

First consider the groups of patients eligible for inclusion in the MYCYC and MYPAN trials.

Mark on the scales below your answers to the following questions (to the nearest 0.05).

Q1: What is the chance that the 6-month remission rate on CYC/steroids in the MYCYC patient group exceeds that in the MYPAN patient group?

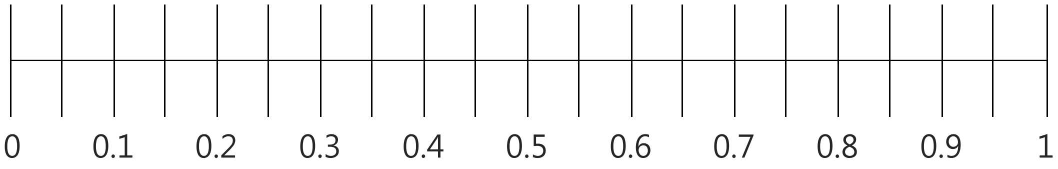

Q2: What is the chance that the 6-month remission rate on CYC/steroids in the MYPAN patient group exceeds that in the MYCYC patient group by more than 10%?

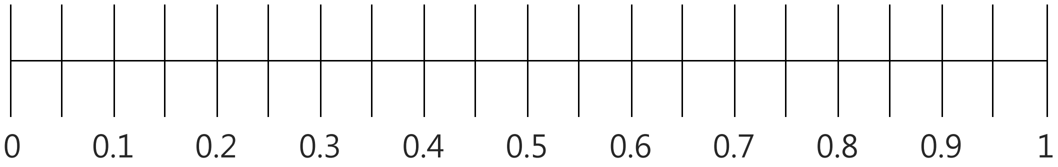

Q3: What is the chance that the 6-month remission rate on MMF/steroids in the MYCYC patient group exceeds that in the MYPAN patient group?

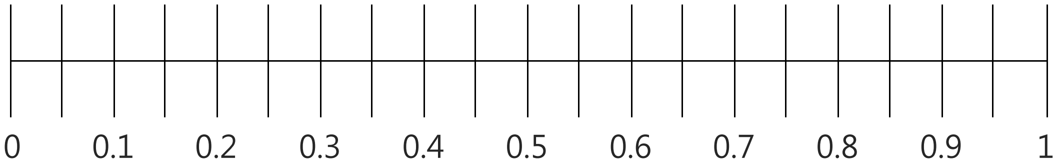

Q4: What is the chance that the 6-month remission rate on MMF/steroids in the MYPAN patient group exceeds that in the MYCYC patient group by more than 10%?

0 0.1 0.2 0.3 0.4 0.5 0.6 0.7 0.8 0.9 1
