# Supplementary material for: Elicitation of Expert Prior Opinion: Application to the MYPAN Trial in Childhood Polyarteritis Nodosa
Source: PLoS One. 2015 Mar 30;10(3):e0120981. doi: 10.1371/journal.pone.0120981 (PMC4378846; doi:10.1371/journal.pone.0120981)
Supplement: S1 Table — (PDF) [file pone.0120981.s003.pdf]

S1 Table: Individual experts' final answers to Q1-Q4 and consensus answers agreed by the group before results from the MYCYC trial were revealed

Expert	Q1	Q2	Q3	Q4
1	0.65	0.45	0.63	0.05
2	0.85	0.60	0.35	0.20
3	0.80	0.55	0.10	0.50
4	0.85	0.65	0.20	0.40
5	0.70	0.60	0.20	0.20
6	0.80	0.80	0.15	0.10
7	0.75	0.50	0.10	0.15
8	0.75	0.55	0.30	0.20
9	0.70	0.60	0.20	0.10
10	0.70	0.60	0.25	0.25
11	0.75	0.55	0.30	0.20
12	0.70	0.50	0.10	0.30
13	0.75	0.40	0.20	0.15
14	0.80	0.55	0.20	0.35
15	0.80	0.60	0.20	0.30
**Mean**	**0.76**	**0.57**	**0.23**	**0.23**
**Median**	**0.75**	**0.55**	**0.20**	**0.20**
**Consensus values**†	**0.70**	**0.50**	**0.30**	**0.30**

Q1: What do you think the 6-month remission rate for children with PAN treated with cyclophosphamide (CYC) in combination with corticosteroids (steroids) is?
Q2: Provide a proportion such that you are 75% sure that the true 6-month remission rate on CYC/steroids exceeds this value.
Q3: What is the chance that the 6-month remission rate on MMF/steroids is higher than that on CYC/steroids?
Q4: What is the chance that the 6-month remission rate on CYC/steroids exceeds that on MMF/steroids by more than 10%?

† Consensus answers to questions were determined by vote. Experts voted for the pair of answers to (Q1, Q2) which they thought best reflected their prior opinion for p_C. Votes were cast between pairs of answers (0.7, 0.5) and (0.75, 0.55), which received 10 (67%) and 4 (27%) votes, respectively; one expert abstained. Consensus answers were those voted for by the majority as reflecting their opinion. Consensus answers to (Q3, Q4) were determined by a similar process, with experts casting votes between the following pairs of answers: (0.3, 0.3) and (0.3, 0.35) which received 12 (80%) and 3 (20%) votes, respectively.
