# Supplementary material for: Elicitation of Expert Prior Opinion: Application to the MYPAN Trial in Childhood Polyarteritis Nodosa
Source: PLoS One. 2015 Mar 30;10(3):e0120981. doi: 10.1371/journal.pone.0120981 (PMC4378846; doi:10.1371/journal.pone.0120981)
Supplement: S2 Table — (PDF) [file pone.0120981.s004.pdf]

S2 Table: Clinical trials in adults with polyarteritis nodosa

Study	Study population	Trial design	Years to recruit	Outcome	Adverse events	Conclusion
**Ribi et al 2010 (A&R 62:1186-1197)**	124 adults with PAN (n=58) or MPA (n=66) with FFS=0	Open-label RCT: 39 patients who failed corticosteroids randomised to receive CYC or AZA. Corticosteroids given to both groups.	12	Remission: 69% CYC<br>70% AZA<br>Death: 20%<br>32% CYC<br>10% AZA	54% : equal in CYC or AZA	AZA or CYC reasonably effective for treating mild but corticosteroid resistant PAN/MPA
**Guillevin et al 2003 (A&R 49: 93-100)**	65 adults with PAN (n=18) or MPA (n=47) with FFS≥1	Open-label RCT of 6 vs 12 intravenous doses of CYC. Corticosteroids given to both groups. AZA not given on cessation of CYC.	6	Remission: 86%<br>Relapses:<br>22% for 12 CYC<br>66% for 6 CYC<br>Death: 22%<br>18% for 12 CYC<br>26% for 6 CYC	100% had nausea/vomiting<br>51% had other side effects	6 CYC pulses are less effective than 12 CYC pulses to treat severe PAN and MPA
**Gayraud et al 1997 (Br J Rheum 36: 1290-1297)**	25 adults with PAN (n=17) or CSS (n=8) with FFS=0	Open-label RCT of daily oral CYC vs monthly-IV CYC	4	Remission: 76%<br>no difference between oral or IV CYC. Higher cumulative CYC dose in oral group<br>Relapses: 16%<br>similar in both groups<br>Death: 4%<br>1 patient in IV CYC group died of sepsis.	72%<br>Oral CYC: 27 side effects in 10/12 patients<br>IV CYC:<br>14 side-effects in 8/13 patients	IV CYC recommended rather than oral CYC based on more favourable therapeutic index: overall lower cumulative dose with comparable efficacy.
**Guillevin et al 1995 (A&R 38:1638-1645)**	62 adults with PAN (n=48) or CSS (n=14) with FFS≥1	Non-blinded RCT of CS-CYC (n=28) versus CS-CYC plus plasma exchange (n=34)	5	Remission: 61%<br>no difference +/- plasma exchange.<br>Death: 18%<br>25% without plasma exchange<br>12% with plasma exchange (NS).	Infection: 15%<br>Lymphoma: 1 patient	Additional plasma exchange is not superior to treatment with CS-CYC alone.

PAN: polyarteritis nodosa; MPA: microscopic polyangiitis; FFS: 5 factor score; RCT: randomised controlled trial; CYC: cyclophosphamide; AZA: azathioprine; CSS: Churg Strauss syndrome; CS: corticosteroid; NS: non-significant.
