# Supplementary material for: Elicitation of Expert Prior Opinion: Application to the MYPAN Trial in Childhood Polyarteritis Nodosa
Source: PLoS One. 2015 Mar 30;10(3):e0120981. doi: 10.1371/journal.pone.0120981 (PMC4378846; doi:10.1371/journal.pone.0120981)
Supplement: S3 Table — (PDF) [file pone.0120981.s005.pdf]

S3 Table: Individual experts' final answers to four questions eliciting their beliefs about the relevance of the MYCYC trial results for informing opinion about 6-month remission rates in the MYPAN trial. Individual and consensus answers were reached after a summary of the design of MYCYC was provided, but before the actual MYCYC results were revealed.

Expert	Q1	Q2	Q3	Q4
1	0.60	0.50	0.65	0.60
2	0.50	0.20	0.50	0.20
3	0.55	0.40	0.50	0.50
4	0.65	0.20	0.35	0.15
5	0.50	0.20	0.40	0.60
6	0.50	0.50	0.50	0.50
7	0.55	0.40	0.55	0.40
8	0.60	0.10	0.60	0.10
9	0.50	0.30	0.30	0.20
10	0.50	0.20	0.50	0.20
11	0.50	0.50	0.50	0.50
12	0.70	0.20	0.60	0.10
13	0.50	0.30	0.30	0.40
14	0.60	0.25	0.25	0.25
15	0.60	0.20	0.60	0.20
**Mean**	**0.56**	**0.30**	**0.47**	**0.33**
**Median**	**0.55**	**0.25**	**0.50**	**0.25**
**Consensus values**†	**0.55**	**0.25**	**0.5**	**0.25**

Q1: What is the chance that the 6-month remission rate on CYC/steroids in the MYCYC patient group exceeds that in the MYPAN patient group?
Q2: What is the chance that the 6-month remission rate on CYC/steroids in the MYPAN patient group exceeds that in the MYCYC patient group by more than 10%?
Q3: As Q1 above but concerning MMF/steroids instead of CYC/steroids.
Q4: As Q2 above but concerning MMF/steroids instead of CYC/steroids.

†Once individuals had finalised their answers to Q1-Q4, median values were calculated and it was proposed that these be taken forward as a working summary of the group's consensus opinion. Once the MYCYC data were revealed, median values of answers to Q1-Q4 were used to incorporate these data into the prior distributions for θ, p_C and p_E based on expert opinion alone. Through constructive discussions, experts then reached the consensus that densities incorporating MYCYC data had face validity for representing their beliefs (93% agreement; no abstentions permitted).
